# A Characterisation and Profiling of District Health Indicators in Zimbabwe: An Application of Principal Component Analysis in a Data Limited Setting

**DOI:** 10.36469/9833

**Published:** 2015-12-04

**Authors:** Shepherd Shamu, Simbarashe Rusakaniko, Charles Hongoro

**Affiliations:** 1 College of Health Sciences, Department of Community Medicine University of Zimbabwe; 2 Human Sciences Research Council of South Africa

**Keywords:** reliability, validity, principal component analysis, composite indicators, equity, cross variation

## Abstract

**Background:** The Ministry of Health and Child Care, Zimbabwe does not have a method for prioritization and equitable allocation of its share of the national health budget and other resources in the sector. Regional allocations at the provincial level are made regardless of the provinces’ disease burden, population size, or needs. Currently there is no method available to show how the provinces eventually allocate these resources to the lower levels of care. In a data limited country such as Zimbabwe, Principal Component Analysis method can be used to identify a set of indicators that account for cross variation between different regions. This set of indicators could then be used by planners as reference indicators for equitable allocation of resources and prioritization of health care interventions.

**Objective:** The aim of the study was to construct a set of simple, feasible, reliable and valid composite health indicators for use in characterising and profiling of the different districts in Zimbabwe.

**Method:** This was a retrospective analysis of secondary data to derive composite indices for the 57 administrative health districts in Zimbabwe using routinely collected secondary data. The data was extracted from the 2012 Zimbabwe Health information database, the 2012 National Census and the 2011 Prices, Income and Expenditure Survey.

**Results:** The analysis of the data resulted in the construction of 10 mutually exclusive principal composite indices, which included demographic, child related, disease related and health systems related indices. The 10 composite indices (population, immunisation, child mortality, antenatal care, HIV/TB, malaria, non-communicable diseases, socioeconomic, health seeking behaviour and infrastructure) were tested for construct and content validity and were found to be statistically robust, reliable and consistent with observed behaviour.

**Conclusion:** The composite indices exhibited internal consistency and construct validity to be regarded as true representations of the cross variation of the 57 districts in Zimbabwe; hence these indices could be used to characterise the behaviour and assess the performance of these districts. There is also potential use for these indices in the areas of resource allocation and prioritisation of health interventions.

## INTRODUCRION

Ministry of Health and Child Care, Zimbabwe does not have a method for prioritization and equitable allocation of its share of the national health budget and other resources in the sector. Regional allocations especially at provincial level are currently done on almost equal basis regardless of the provinces' disease burden, population size, or needs. Currently there is no method or data available to show how the provinces eventually allocate these resources to the lower levels of care. In a data limited country such as Zimbabwe, a Principal Component Analysis (PCA) method can be used to identify a set of indicators that account for cross variation between different regions in Zimbabwe.

The PCA method is not a new method, it has been used in number of studies for classifying different geographic areas, disease groups and for analysing patterns of different dietary behaviours for different communities.[1] The main aim of the study was to construct a set of simple, feasible, reliable and valid public health system indices for use in characterising and profiling of the 57 different districts in Zimbabwe.

This set of composite health indices have future potential use by policymakers and planners as baseline reference indices for equitable allocation of resources and prioritisation of health care interventions. These indices could then be refined over time as more and more complex data are introduced.

To achieve this objective, we used PCA method to construct a set of few indicators that are not correlated and are able to explain the largest variation in the data. These indicators can either be retained as they are or the originally correlated indicators can be used to build uncorrelated and independent composite indicators from a linear combination of their constituent indicators.[2] We took the second option of constructing independent composite indicators from sets of correlated health indicators. Composite indicators have the intuitive appeal of providing a summary of indices for often complex and multi-dimensional issues.[3]

A number of studies have used PCA in spatial analysis involving multi-dimensional health indicators. PCA is commonly used in the construction of socioeconomic quintiles from Demographic Health Surveys for monitoring of equity of different population groups.[2,4] In 2002 McIntyre et al[5] used PCA to construct a general index of deprivation for South Africa. They used the index to assess the relationship between socioeconomic status and health so as to inform resource allocation in the health sector in different provinces of South Africa. In particular, PCA was more useful in the identification of variables that could possibly explain some of the small pockets of deprivation in South Africa. One important finding of their study was the feasibility of using PCA for small area analysis even in data limited contexts. Sun et al[6] explored the use PCA and other methods in assessing the health impacts of environmental factors in a multipollutant model. In all these studies, PCA proved to be a robust and useful method for spatial analysis.

## METHODOLOGY

This was a retrospective analysis using secondary data that was extracted from the 2012 Zimbabwe Health information database, the 2012 National Census[7] and the 2011 Poverty, Income, Consumption and Expenditure Surveys.[8] Using expert opinion, we scanned literature and the local health information databases for the selection of relevant indicators and identified an initial set of 40 individual indicators.[3,9,10] These were selected on the basis that they at least represented the districts' demographic, disease, health system and socioeconomic status.

The study sample comprised of 57 districts out of a total of 60 districts in the country whose primary focus is the provision of primary health care. Gokwe South and Gokwe North districts were combined into one district, while Harare and Bulawayo districts were excluded because they provided a lot of secondary and higher level care. The study used the PCA approach to derive the resultant composite indicators for the 57 districts.

### Domain Clustering and Colour Coded Data Visualisation

While Lindman and Sellin[11] noted that in most cases the principal components and their resultant index scores may not be readily interpretable, a more focused grouping of variables into more relevant and common domains can make the resultant index scores more meaningful and readily interpretable. To circumvent the difficult of interpretability of the index scores, we had an a priori domain clustering of the initial 40 indicators that we started with, grouping indicators and putting indicator with similar themes into domains.

We grouped a set of initial 40 variables into 10 domains as shown in Table 1, based on correlations of constituent key indicators, availability of key indicator data, easiness of collection of the key indicators, and the quality of those indicators for all the 57 districts.

We used a technique called heatmap which can give a clear visual picture of correlated indicators to aid in the initial domain clustering of indicators. Figures 1a-d below show an example of how we used the heatmap technique to cluster the child immunisation indicators. Using this technique we substituted numbers with colours to display some visual pattern of the original data set. Using Quantum GIS software for mapping, we used a colour coded mapping technique to visualise the correlation of selected child immunisation indicators (Measles, Oral Polio Vaccine [OPV], Bacillus Calmette–Guérin [BCG] and Pentavalent) in terms of their coverage in the 57 districts. A visual comparison of the four maps below shows the coverage of the immunisation indicators was not different for all the 57 districts as reflected by the same spatial colour codes in the different parts of the heatmaps. It therefore meant that either one of the immunisation indicators could be used to show cross variation across the districts or the four indicators could be combined to construct a single composite indicator as they are correlated and would give a richer and more informative composite indicator. In our study we combined the four indicators to construct a more robust composite indicator.

We used this form of heatmap technique for the other domains to complement the literature review and expert opinion survey for a priori domain clustering of indicators. The indicators making up these established domains were then standardised using the Z-score, making it possible to interpret the scores based on the deviation of the index score from the overall group mean.

**Figure 1a-1d.** Immunisation Domain Clustering Using Heatmap Technique

*Figure 1a. Measles Coverage*

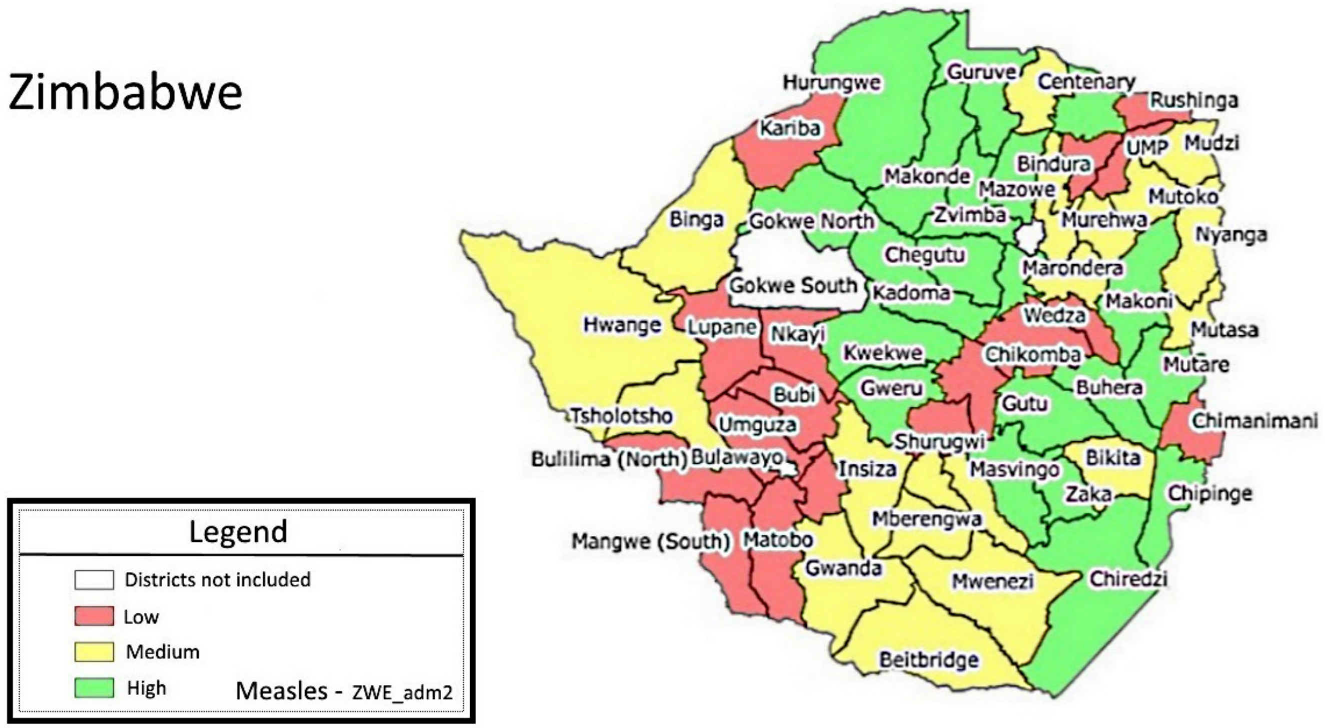

*Figure 1b. BCG Coverage*

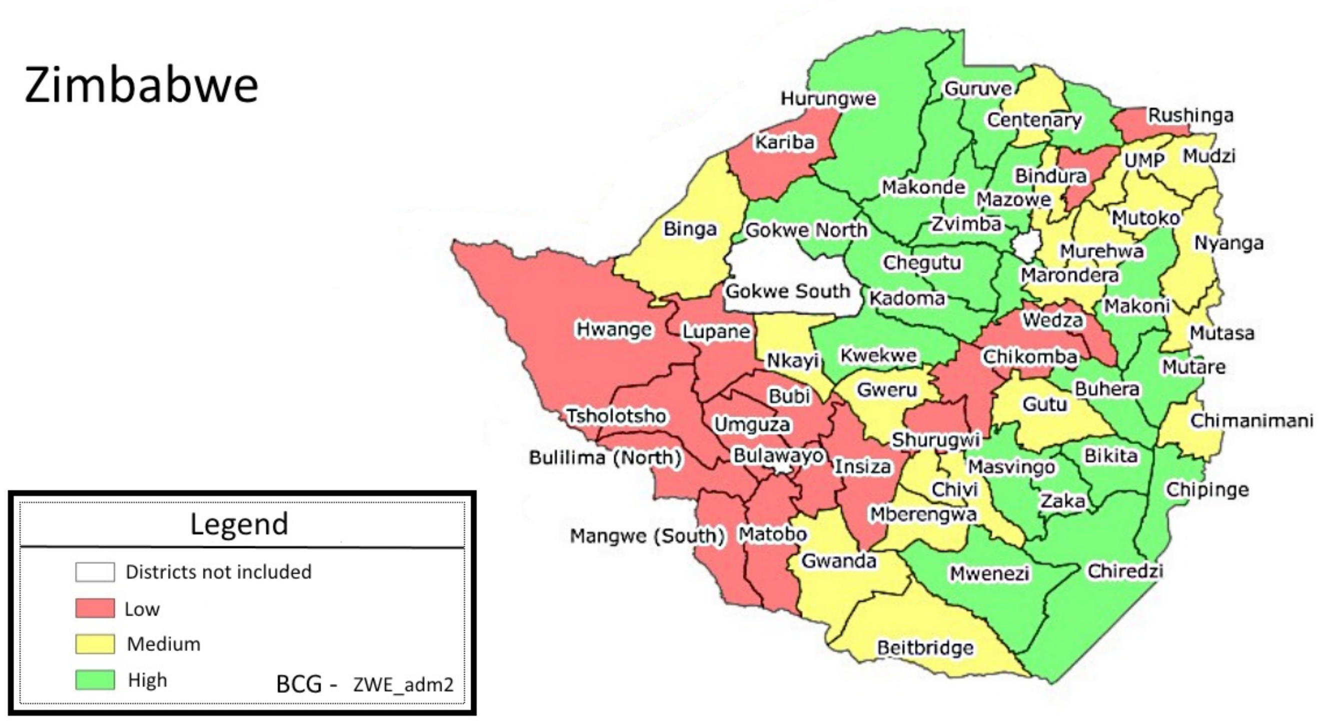

*Figure 1c. Oral Polio Vaccine Coverage*

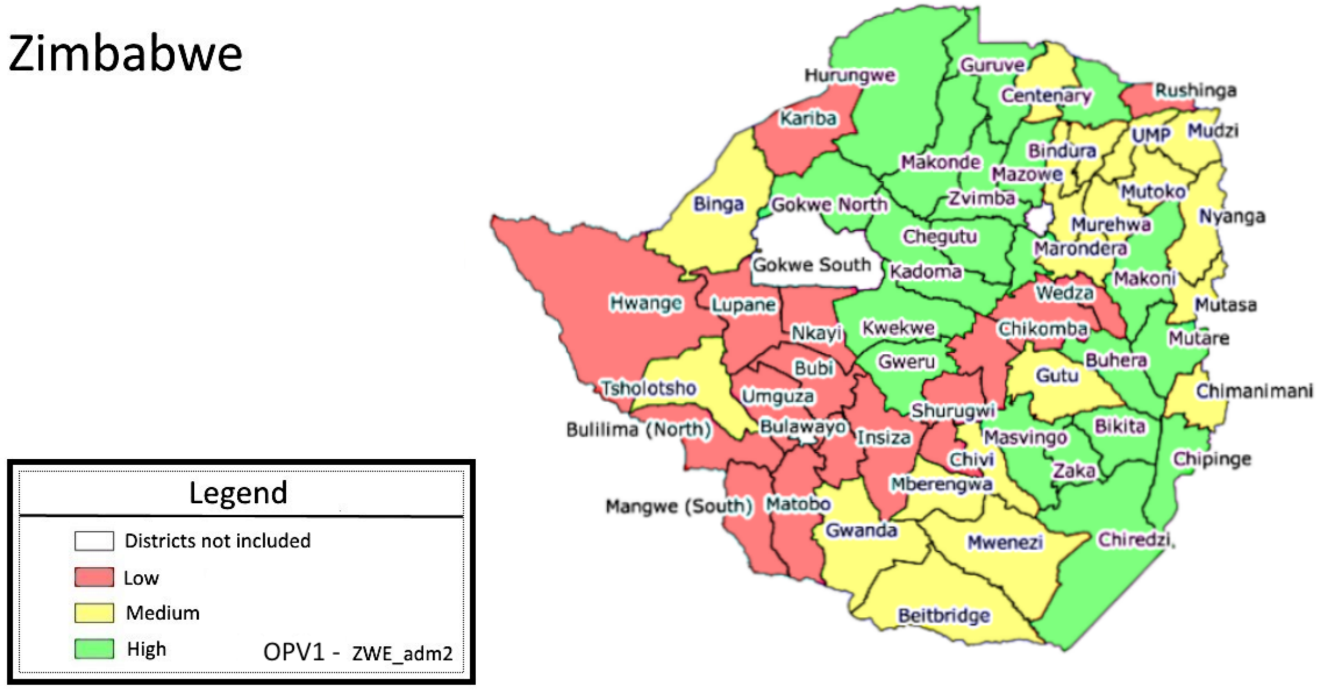

*Figure 1d. Pentavalent Coverage*

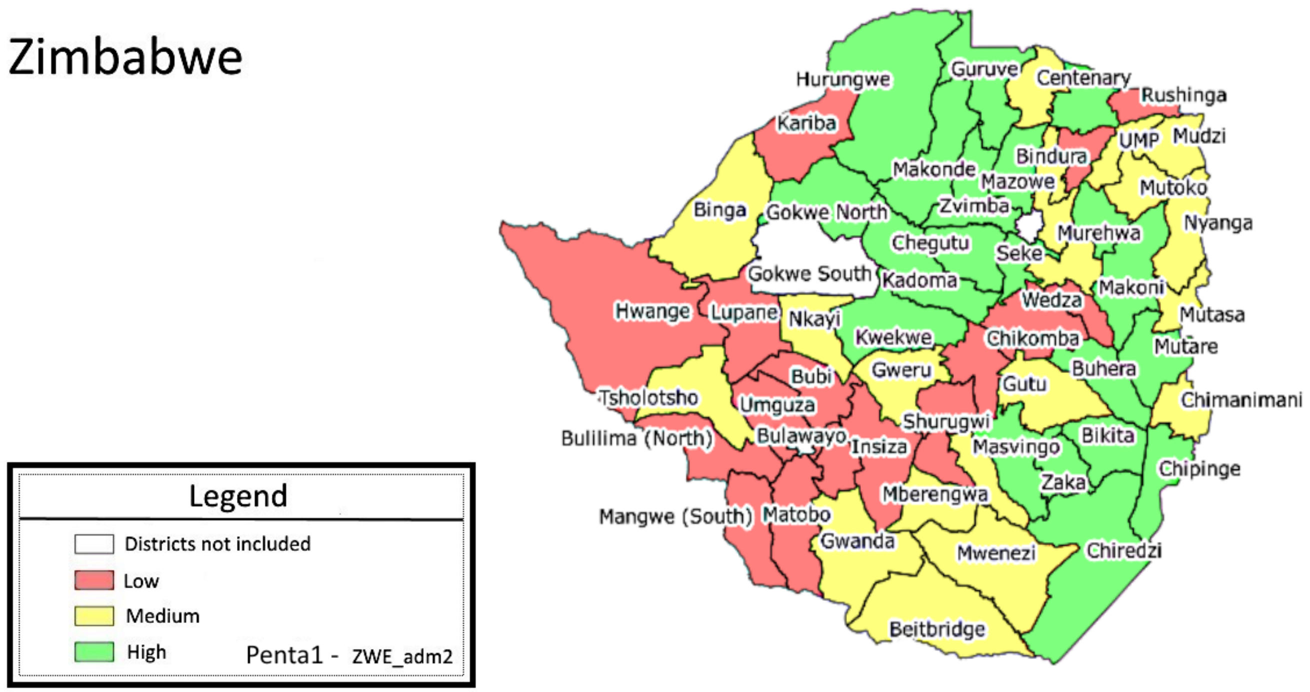

**Table 1.** Domains and Constituent Variables

| Domain | Variables (extracted per district) | Source |
|---|---|---|
| **Immunisation** | Measles % coverage, oral polio vaccine (OPV1-3) % coverage, Bacillus Calmette Guerin (BCG) % coverage, Pentavalent 1-3 % coverage | ZDHS 2010/11; Ministry of Health and Child Care Health information Database 2012 |
| **Health Seeking Behaviour** | Public visits (% share of total), private visits (% share), traditional and faith healers (% share), no visits at all (% share) | Prices Incomes Consumption and Expenditure Survey - Zimstat 2011/12 |
| **Socioeconomic status** | Unemployment (%), poverty status (%), household size (number of family members in a household) | Poverty Survey Report - Zimstat 2011/12 |
| **Non-Communicable Diseases** | Diabetes (number of males and females) Blood Pressure (number of males and females), number of injuries | Ministry of Health and Child Care Health Information Database 2012 |
| **Antenatal Care (ANC)** | Child live birth, live female birth, average number of ANC visits | ZDHS 2010/11; Ministry of Health and Child Care Health information Database 2012 |
| **Child Mortality** | Rate of Under 5 mortality, rate of infant mortality, under 5 weight (%) | ZDHS 2010/11; Ministry of Health and Child Care Health information Database 2012 |
| **HIV/Tuberculosis (TB)** | Number of HIV infected persons, Number of female TB/HIV persons, Number of male TB/HIV persons, number of mothers on ARVs | Ministry of Health and Child Care Health information Database 2012; National AIDS Council Annual Report 2012 |
| **Malaria** | Number of positive Malaria tests (male and female) | ZDHS 2010/11; Ministry of Health and Child Care Health information Database 2012 |
| **Infrastructure** | Number of beds, ratio of curative to preventive services (share of total expenditure), number of health facilities | Ministry of Health and Child Care Health Information Database 2012; Ministry of Health and Child care National Infrastructure Report 2012 |
| **Population** | Total under 5 population, total female population, total district population | Zimbabwe Population Census 2012 - Zimstat |

ZDHS: Zimbabwe Demographic and Health Survey; ARV: antiretroviral

### Construction of the Composite Indicators

Each of the 10 domains had 3 or more variables (Table 1) that were used to construct the composite domain indices using the PCA technique. Using SPSS statistical package we ran a PCA to construct composite indicators that characterised and profiled the 57 districts of interest. We constructed 10 composite indicators for all the identified 10 principal domains. The domain indicators were developed as linear composite indicators of the various constituent indicators *as represented in the following maximum variance linear function of n indicators in the matrix x;*

$$D_{ij} = W_1 x_{1j} + W_2 x_{2j} + W_3 x_{3j} + \ldots\ldots .. + W_n x_{nk}$$

Where $D_{ij}$ referred to the composite indicator for domain $i$ for the *jth* district, $x_{ij}$ referred to the value of the *ith* variable for the *jth* district and $W_i$ referred to the corresponding weight of the *ith* variable.

Each coefficient vector $W_1 x_{1j}$ generated a combination on $W_1 x_{1j}$ which yielded a new composite indicator $D_{ij}$ of $m$ dimensions in an $n$ dimensional space. Each composite indicator for each domain $D_{ij}$ maximised Variance ($X_{ij}\ W_{ij}$) subject to the constraint that the weights ($W_1$) sum to $1$, and covariance ($X_{ij}\ W\ X_{nj}\ W$) equalled 0. The procedure was repeated for all the 10 domains to create a system of maximum variance of uncorrelated linear composite indicators representing the 10 domains. The first PCA generated different weights for the indicators by assigning them the components from the first eigenvector of the covariance matrix. In our analysis we only focused on the first principal component since weights from the first PCA are commonly used to create composite indicators.[2] For each domain, the first principal component with the largest eigen value was retained as the composite domain indicator of interest. When indicators are measured using the same scale as was the case in this study, the PCA with covariance matrices gives the greatest weight to the indicator with a numerically higher variance; hence each principal component's weight was based on the explanatory power of its main indicators. Of interest, the standardisation of the correlation matrices enabled easier analysis and comparison of data sets presented in different units.[11,12,13] Indicators with higher variation across districts loaded more weight on the domain indicators, enabling more visible variation across the districts.

## RESULTS

Our results were analysed based on how our final composite indicators met the key conditions of robustness, reliability and empirical relevance. This was done using an analytic framework that assesses for construct and content validity of the new composite indicators.

### Construct Validity

While the definition for construct validity may differ, Carmine and Zeller[14] refer to it as meaning that concepts are clearly defined and justified. Our PCA analysis of the data resulted in the construction of 10 mutually exclusive principal composite indices as shown in Table 2, passing the first test of construct validity.

**Table 2.** Results of the Principal Component Analysis

| Domain Index | Variables | Determinant | K-M-Oklin | Bartlett's test of Sphericity; Chi-square (significance) | Lowest Anti-image Correlation | Total Variance (%) | Cronbach's Alpha if Domain Index is Deleted from Scale |
|---|---|---|---|---|---|---|---|
| **Immunisation** | Measles, OPV1-3, BCG, Penta1-3 | 1.4 | 0.814 | 1430.6 (.000)*** | 0.725 | 95.89 | .473 |
| **Health Seeking Behaviour** | Public visits, private visits, traditional and faith healers, no visits at all | 0.05 | 0.670 | 21.68 (.000)*** | 0.502 (none variable had an anti-image/ sampling adequacy of 0.4; hence was dropped) | 54.8 | .551 |
| **Socioeconomic Status** | Unemployment, poverty status, household size | 0.547 | 0.650 | 32.69 (.000)*** | 0.62 | 63.5 | .714 |
| **Non-Communicable Diseases** | Diabetes (males and females) Blood Pressure (male and female), injuries | 0.145 | 0.711 | 103.85 (.000)*** | 0.66 (injuries variables had an anti-image/ sampling adequacy of 0.18; hence was dropped) | 67.5 | .670 |
| **Antenatal Care (ANC)** | Live birth, live female birth, ANC visits | 0.592 | 0.536 | 28.36 (.000)*** | 0.523 | 56.8 | .638 |
| **Child Mortality** | Under 5 mortality, infant mortality, under 5 weight | 0.73 | 0.73 | 17.07 (.000)*** | 0.555 | 54.32 | .504 |
| **HIV/TB** | HIV infected, Female TB/HIV, Male TB/HIV, ARV mothers | 0.017 | 0.535 | 218.53 (.000)*** | 0.52 | 57.13 | .536 |
| **Malaria** | Positive Malaria tests (male and female) | 0.012 | 0.5 | 241.06 (.000)*** | 0.5 | 99.7 | .596 |
| **Infrastructure** | Number of beds, ratio of curative to preventive services, number of health facilities | 0.942 | 0.5 | 3.233 (.007)*** | 0.5 | 62.0 | .575 |
| **Population** | Under 5 population, female population, total population | 5.5 | 0.79 | 655.75 (0.000)*** | 0.734 | 99.9 | .485 |

***0.001

All the 10 composite indicators were found to be significant at 0.05. The ratio of cases (districts) to variables (composite indicators) was 5.7: 1, while all the composite indicators explained more than 50% of the variation.

### Internal Consistency Reliability

In general if factors are highly correlated, then the resultant domain factor or principal component would be regarded as reliable. We selected the first principal component, which was a linear combination of the different variables for each respective domain. For each domain we retained the principal component if it accounted for more than 50% of the variation and had a communality of greater than 0.5. The overall Cronbach's alpha for the 10 domain indices was 0.711, which was higher than the recommended threshold of 0.7 and hence showed high levels of internal consistency. The last column in Table 2 shows the value that Cronbach's alpha would be if that particular domain index is removed. We observed that the removal of any domain index, except for the Socioeconomic Index, would result in a lower Cronbach's alpha. We also retained the first PCA on the basis that our final composite indicators fulfilled all the assumptive conditions shown in Table 3 below.

**Table 3.** A Comparison of Assumptive Conditions and the Actual Data for Testing for Internal Consistency

| Condition for Internal Consistency | Actual Results |
|---|---|
| Sample size of not less than 50 | 57 districts |
| Ratio of cases to indicators of more than 5: 1 | 5.7 : 1 |
| Correlation matrix for the indicators contained 2 or more correlation of 0.30 or greater | >0.4 - Note that 0.4 is used as a heuristic value.15 Tabachnick and Fidell16 suggested a value of 0.3 or greater. |
| Individual indicators had a sampling adequacy of not less than 0.5 | >0.5 |
| Sampling adequacy of 0.5 or more for the Principal Components | >0.5 |
| Non-Zero determinant | > 0 |
| Bartlett's test of sphericity for the principal components | All significant at 0.05 |

### Stability of the PCA

Stability of PCA refers to the degree of sensitivity of the analysis to variations in data and model parameters.[17,18] The PCA can be regarded as stable if a small, unimportant change in data leads to a small, unimportant change in the results.[18] While the stability of a multivariate PCA can be tested using the assumptions of normality, for purposes of sensitivity analysis, we also ran a non-parametric bootstrap procedure to test for the stability of the principal components.[18,19,20] Non-parametric bootstrapping is a procedure where one draws indicators randomly with replacement, that is X bootstrap samples from the original dataset and compare the different principal components. We found the coverage percentages of the bootstrap percentile confidence regions to be significant with a probability of 99%. We then used the Friedman test to test for differences and variations of the principal components across the 57 districts. We tested the null hypothesis that the distribution of the scores in each component and across the 57 districts was the same. We found no evidence that the principal components were dependent of each other; hence were significantly different across the 57 districts (chi-square of 2.38, p-value $< 0.01$). It meant therefore that our composite indicators were statistically independent and could be used to characterise and profile the districts and show cross variation across them. It also meant that if one were to rank the districts using each one of the 10 composite indicators, one would get different rankings for each district. A p-value of $< 0.05$ for the Bartlett's test of sphericity, also showed that the principal components improved as the eigenvalues increased hence proved the stability of the 10 composite indicators.

**Table 4.** Ranking Test for the 10 Composite Indices

| Composite Index | Rank |
|---|---|
| Immunisation Index | 5.33 |
| Health Seeking Behaviour Index | 5.61 |
| Socioeconomic Index | 5.56 |
| Non-Communicable Disease Index | 5.32 |
| Antenatal Disease Index | 5.86 |
| Child Mortality Index | 5.68 |
| HIV/TB index | 5.35 |
| Malaria Index | 5.21 |
| Infrastructure Index | 5.67 |
| Population Index | 5.39 |

Chi-square 2.38, Monte Carlo Significance = 0.985 and Confidence Interval = 0.982-0.988

## Criterion or Concurrent Validity

We also assessed ways in which the results were consistent with observed relationships, prior research and experience on the ground. Using spatial distribution maps for selected composite indicators we profiled the 57 districts by assessing how the selected composite indicators measured against the observed behaviour of the main loading indicators. We assessed whether the resultant composite indicators gave a different picture from the observed reality on the ground. The spatial distribution of the immunisation index (Figure 2a) shows the deviations of the district indices from the group average. The higher the deviation was from the group mean the better and more the immunisation coverage was. A comparison of the composite indicator to the individual immunisation indicators (Measles, BCG, OPV and Pentavalent) showed changes in the ranking and positions of districts indicating the advantages of using a composite indicator against single indicators. The composite indicator offered better information than a single indicator. The same pattern was also observed for the other composite indicator on Infrastructure (Figure 2b). Districts that had a better socioeconomic status also had better health outcomes overall; hence our theoretical composite indicators did not differ significantly from observed behaviour on the ground.

**Figure 2a.** Spatial Distribution of the Immunisation Index

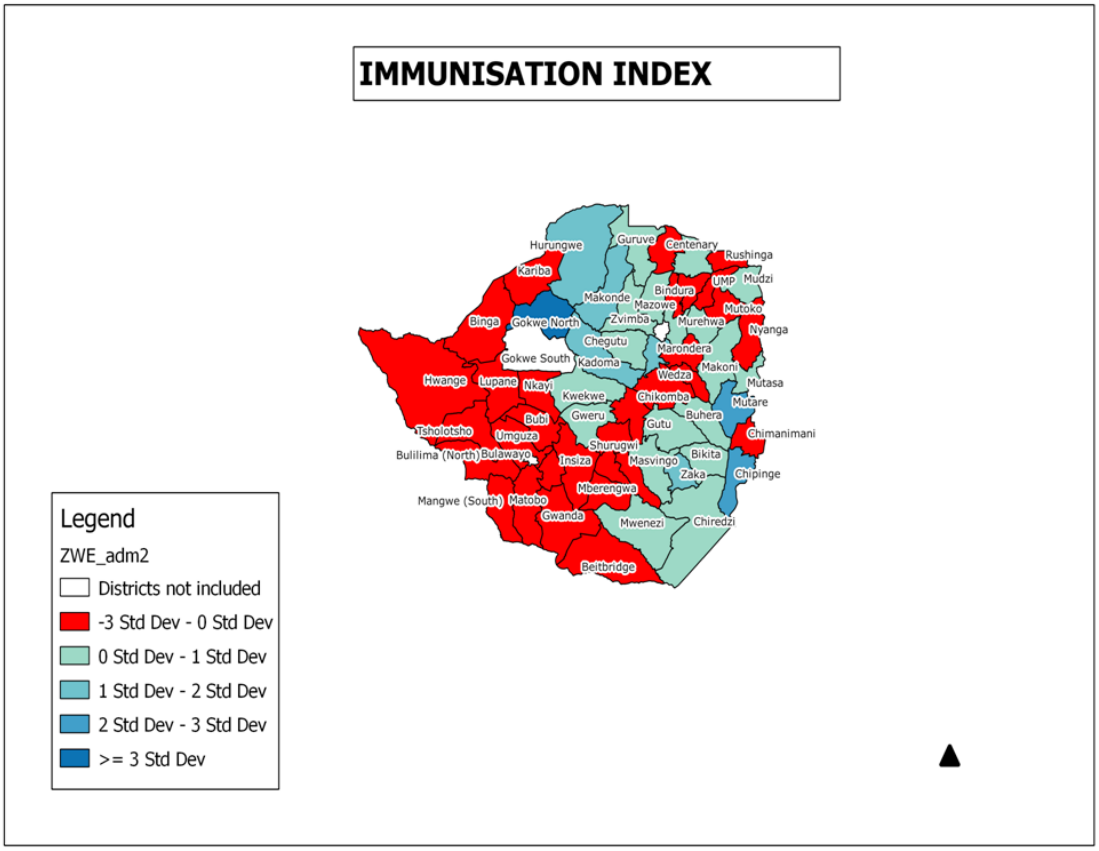

**Figure 2b.** Spatial Distribution of the Infrastructure Index

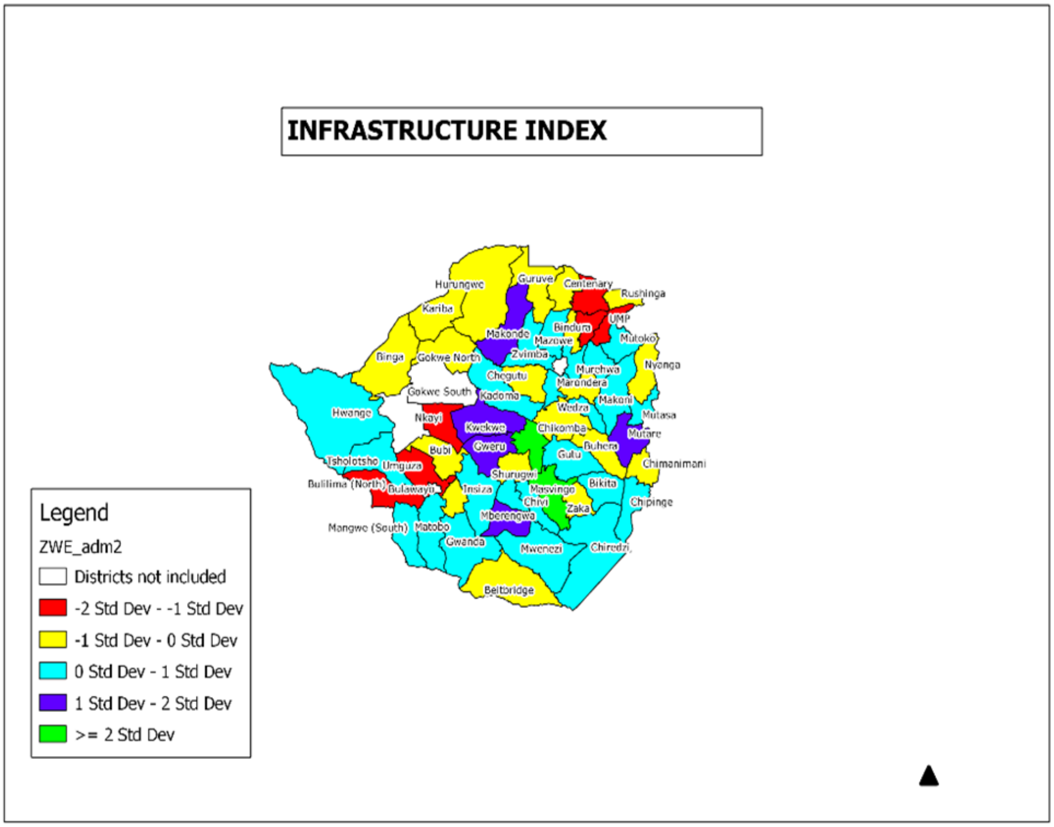

Using scatterplots we also assessed for the construct validity of the following composite indicators; socioeconomic index, health seeking behaviour index, non-communicable disease index, antenatal care, child mortality index and the infrastructure index against a health outcome of interest. We tested for the hypothesis that the theoretical composite indicators conformed to the observed behaviour of the 57 districts in terms of the relationship between health outcomes and socioeconomic status. In general and from empirical evidence, higher socioeconomic status is normally associated with better health outcomes.

Table 5 summaries the general trend in the relationship between the districts' socioeconomic status and selected health outcomes.

**Table 5.** Relationship between Selected Indices

| Indicators | Association (+/-) | Explanation of Trend |
|---|---|---|
| **SES/Immunisation** | + positive | The higher the district's socioeconomic status the higher the immunisation rates |
| **SES/Health Seeking Behaviour** | + positive | The higher the socioeconomic status the higher the district's health seeking behaviour |
| **SES and Child mortality** | - negative | The higher the district's socioeconomic status the lower the child mortality |
| **SES and NCDs** | + positive | The higher the district's socioeconomic status the higher the number of people with noncommunicable diseases |
| **Infrastructure and child mortality** | - negative | The higher the infrastructure index the lower the child mortality |

SES: Socioeconomic Status; NCDs: Non-Communicable Diseases

We provide below a few examples of scatterplots which show intuitive relationships between socioeconomic status and the following indices: health seeking behaviour, non-communicable diseases and antenatal care.

The scatter diagram on socioeconomic status and health seeking behaviour (Figure 3) shows a positive relationship, indicating that districts with better socioeconomic status have a higher health seeking behavior index. However, there are also atypical districts such as Kariba and Beitbridge where there is better socioeconomic status but lower health seeking behaviour. Such an observation would then necessitate a further investigation of such districts to understand why they have better socioeconomic status but poor health seeking behaviour.

**Figure 3.** Scatterplot of Socioeconomic Index and Health Seeking Behaviour Index

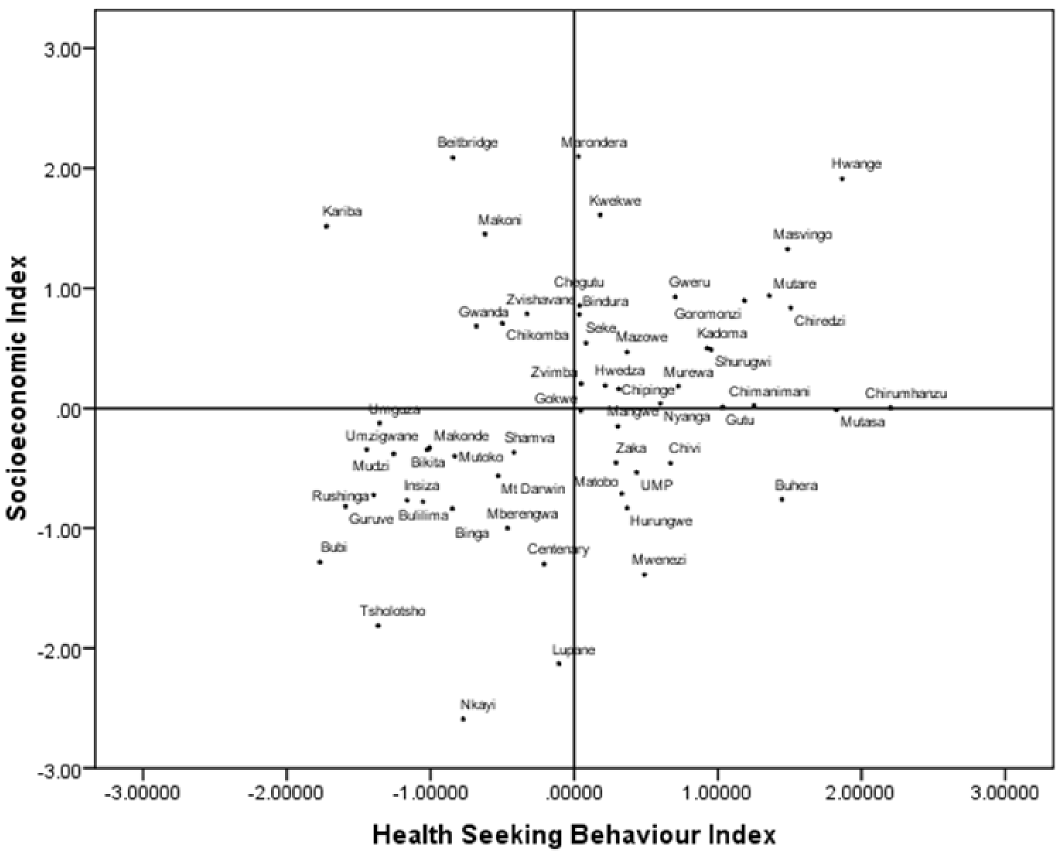

There was also an interesting relationship between socioeconomic status and the non-communicable diseases index (Figure 4). Districts which lie in the upper right quadrant have higher socioeconomic status and higher burden of non-communicable diseases, which is somehow in conformity with the general perception of NCDs being positively associated with affluent societies.

**Figure 4.** Scatterplot of Socioeconomic Status Index and Non-Communicable Diseases Index

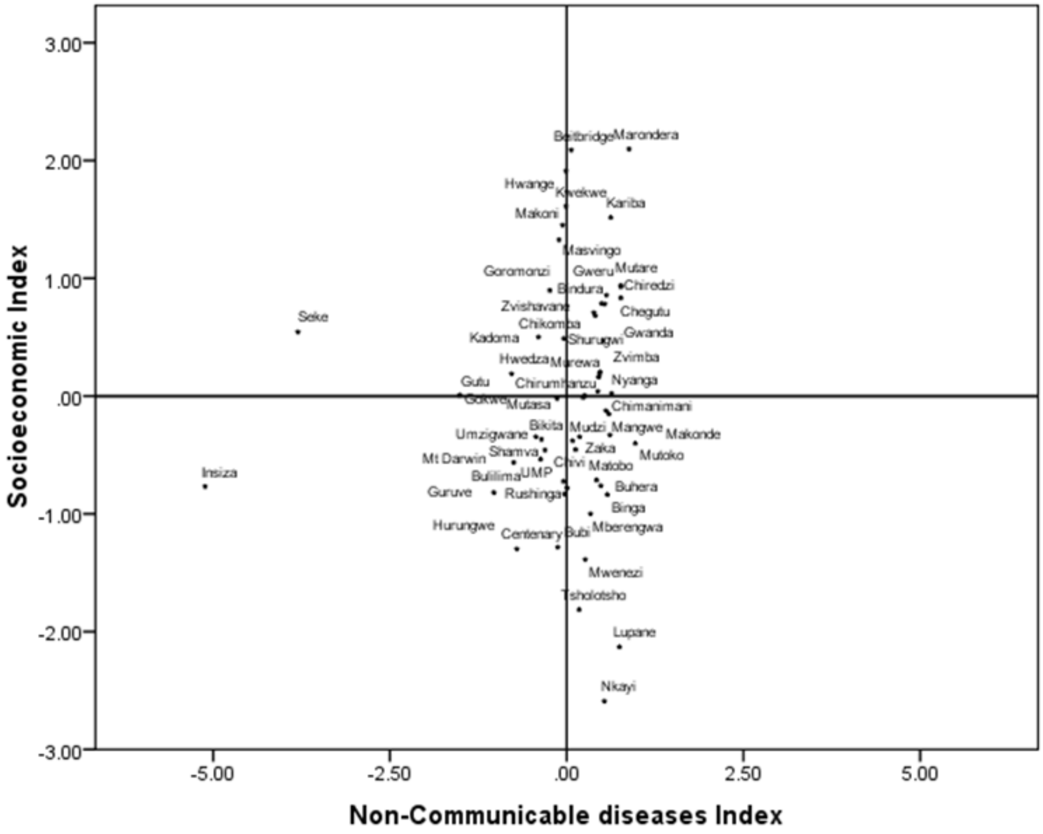

The scatter plot for antenatal care and socioeconomic index (Figure 5) shows a clustering of the districts in the centre of the 4 quandrants, indicating little differences across the districts and probably signifying the influence of the provision of free maternal and child health services. Since 2011, the Health Transition Fund, a multi-donor pooled funding initiative and the World Bank's Results Based Financing Programme, administered by UNICEF and Cordaid respectively, have been providing funds for the provision of free maternal and child health care.

**Figure 5.** Scatterplot of Socioeconomic Index and Antenatal Care Index

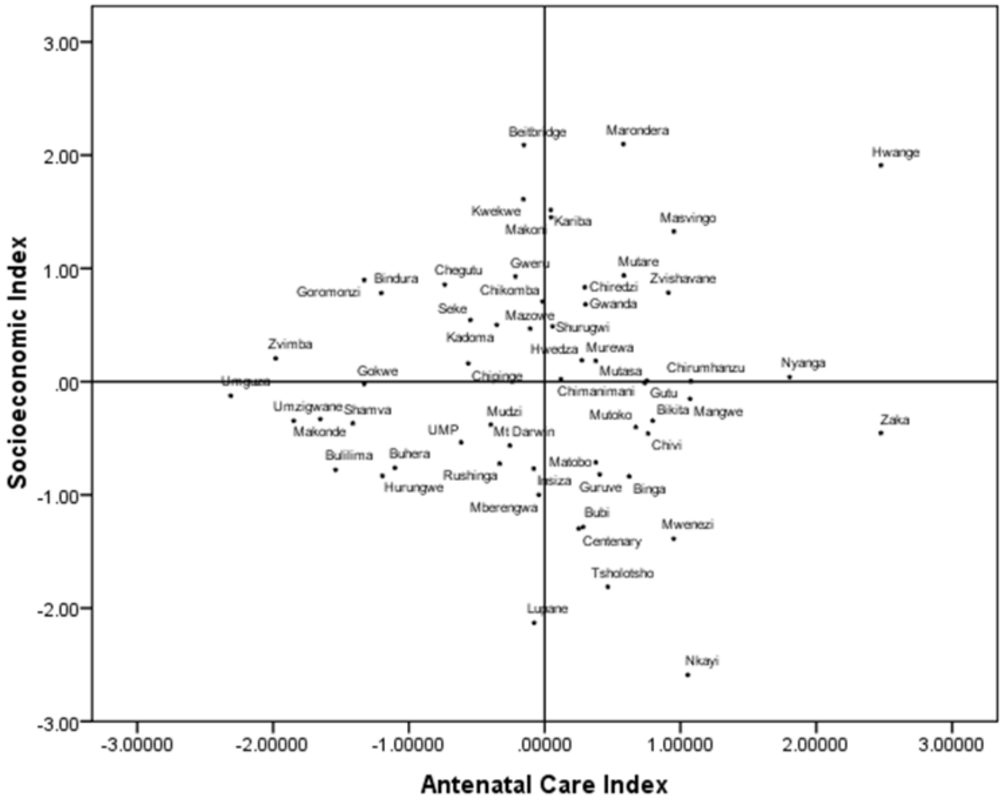

## DISCUSSION

The PCA technique enabled us to construct 10 composite indicators that are robust and reliable. The indicators showed cross variation in health outcomes, health status and socioeconomic status across all the sampled 57 districts. These 10 composite indicators provided a more intuitive understanding of the status of districts than a single indicator or a huge array of often complex and multidimensional indicators. Using PCA method we were able to reduce an initial long list of indicators to a short list of relevant and manageable set. We also found the heatmap GIS mapping technique and key informant interviews very useful in clustering indicators into relevant and more intuitive domains. The World Bank also uses initial clustering by thematic area for its Doing Business indicators as a way of constructing relevant and more intuitive composite indicators.[3]

We found the validation technique used by Smylie et al[21] in the construction of their sexual health indicators more plausible for our study. Their validation technique looked at content validity (factor structure, internal consistency reliability and stability of the factor structure) and construct validity of the principal components for the construction of sexual health indicators for Canadians aged 16-24 years. Just as in our case their validation technique assessed the relationship between their composite indicators and the generally observed behaviour of the constituent indicators and found them to be consistent. McIntyre et al[5] noted that while the PCA technique was a purely statistical method, it could be used to construct potential indicators that are conceptually relevant for assessing deprivation. Bell et al[22] also proposed the use of Geographic Information System for the construction of a deprivation index. In our analysis we showed that both the heatmap GIS technique and the PCA method and GIS can be used for the construction of valid, robust and geographically unbiased indicators which have a more intuitive use in areas such as resource allocation and prioritisation of health interventions. PCA analysis has many applications in social and physical sciences, and in spatial analysis such as the characterisation of different geographic settings.[23] In our study, we were able to show cross variation in health indicators across the 57 geographical districts in the country. We also constructed a socioeconomic index, which is conceptually similar to the wealth index that is generally used in health equity analysis. According to Chakraborty et al[4] the wealth index that is normally constructed using the PCA technique is regarded as valid and reliable in the interpretation of socioeconomic status. The wealth index first constructed by Filmer and Pritchet using PCA and data from India has become the internationally recognised method for constructing a household wealth index using data from Demographic Health Surveys (DHS).[24] Krefis et al[25] used PCA to analyse the socioeconomic factors and its relationship with malaria in children in Ghana. The PCA method enabled the researchers to show variation among households by socioeconomic status.

While in literature some authors have argued for the use of single indicators which are easier to monitor than composite indicators, we believe constructing composite indicators provides a better and more intuitive appeal.[5] Krefis et al[25] noted that the use of single indicators for analysing risk often resulted in false conclusions. We also believe that having more composite indicators as compared to a single composite indicator increases cross variation and plausible comparisons across districts.

In order to test the argument that a single composite indicator hides important information we tried to reduce the number of composite indicators from 10 to only 4 by merging them. While the 4 composite indicators retained accounted for about 75.8% of the total variation, the composite indicators that resulted showed huge variation from observed behaviour and the actual data representation on the ground. Some indicators loaded more than once on the principal components exhibiting some form of complex structure. We therefore decided to retain all of the previous 10 composite indicators as more representative. Our conceptual proposal for the use of more composite indicators may be understood from the analytic work on the usefulness of the asset index as a single composite indicator that was done by Sharker et al.[26] Using simulations they found out that the single asset index had a more than 50% chance of misclassifying wealth quintiles, and that the index itself explained less than 30% of the variance in the component variables.

### Limitations of the Study

The principle behind the construction of composite indicators is a well developed area, however the use of such indicators in decision making is normally affected by the non-availability of routine and reliable data in most developing countries. In our study, in the absence of routine data we made use of survey data, however survey data is not collected on an annual basis; hence may have some lag effect which may affect the validity and reliability of the resultant composite indicators. Other important indicators such as Gross Domestic Product (incomes, expenditures or consumption), gini coefficient and the Human Development Index are not disaggregate by district; hence were left out in the construction of the composite indicators.The inclusion of perception-based indicators based on public opinions and private preferences and health service indicators such as workload indicators would have strengthened our composite indicators.

## CONCLUSION

The composite indicators showed internal consistency and construct validity to be regarded as a true representation of the cross variation of the 57 districts in Zimbabwe. It is important to note that more composite indicators as opposed to an individual indicator or single composite indicator method enabled us to show more informative and intuitive differences across the districts. Composite indicators can be used by the Ministry of Health and Child Care for resource allocation and prioritisation of health interventions in various districts of Zimbabwe. The individual indicators that were used to construct the composite indicators are easily accessible from the country's routine health information system and the numerous population and health surveys that are carried out frequently in the country.

**Acknowledgements**

We would like to thank the Research Support Centre (RSC) staff, Southern African Consortium for Research Excellence (SACORE), Training and Research Support Centre (TARSC), and the Human Sciences Research Council (HSRC) of South Africa for all their support. We also want to thank the Ministry of Health and Child Care (MoHCC) Zimbabwe for providing access to their databases.

**Funding and Declaration of Competing Interests**

This research was supported with funding from the Wellcome Trust (UK) through SACORE and the Research Support Centre, College of Health Sciences, University of Zimbabwe. The authors declare that they have no competing interests

## References

[1] Rao M.V.V, Kumar S, Brahmam GNV: A study of the geographical clustering of districts in the Uttar Pradesh using nutritional anthropometric data of preschool children. *Indian J Med Res* 2013;137(1):73-81.

[2] Vyas S, Kumaranayake L: Constructing socio-economic status indices: how to use Principal Components Analysis. *Health Policy Plan* 2006;21(6):459-68.

[3] Foa R, Tanner J: Methodology of the indices of social development. Harvard and Santa Monica: http://www.indsocdev.org/resources/Methodology%20of%20the%20Social%20Development%20Indices_%20jan11.pdf.

[4] Chakraborty NM, Firestone R, Bellows N: Equity monitoring for social marketing: use of wealth quintiles and the concentration index for decision making in HIV prevention, family planning, and malaria programs. *BMC Public Health* 2013;13(Suppl 2):S6.

[5] McIntyre D, Muirhead D, Gilson L: Geographic patterns of deprivation in South Africa: informing health equity analyses and public resource allocation. *Health Policy Plan* 2002;17(Suppl 1):30-9.

[6] Sun Z, Tao Y, Li S, et al: Statistical strategies for constructing health risk models with multiple pollutants and their interactions: Possible choices and comparisons. *Environ Health* 2013;12(1):85.

[7] ZIMSTAT: Census 2012 National Report. ZIMSTAT: Harare; 2013

[8] ZIMSTAT: Prices, Income, Consumption and Expenditure Report 2011/2012. ZIMSTAT: Harare; 2013

[9] Salzman J: Methodological choices encountered in the construction composite indices of economic and social well-being. Center for the Study of Living Standards. Ottawa; 2003.

[10] Booysen F: An overview and evaluation of composite indices of development. *Social Indicators Res* 2002;59(2):115-51.

[11] Lindman C, Sellin J: Measuring human development. The use of principal component analysis in creating an environmental index. Bachelor Thesis. Uppsala University, Department of Statistics; 2011.

[12] Jolliffe I: Principal Component Analysis. New York: Springer – second edition; 2002.

[13] Kamanou G: Combining development indicators using an Iterative-Principal Component Analysis. United Nations Statistics Division, Joint statistical meetings - Section on Government Statistics. http://www.amstat.org/sections/srms/Proceedings/y2002/files/JSM2002-001154.pdf. Accessed June 29, 2015.

[14] Carmines EG, Zeller RA: Reliability and validity assessment. Sage University Paper Series on Quantitative Applications in the Social Sciences, Sage Publications: London; 1979.

[15] Krishnan V: Constructing an area-bases socioeconomic index: A principal component analysis approach. Early Childhood Council Annual Conference 2010: Christchurch New Zealand; 2010.

[16] Tabachnick BG, Fedell LS: Using multivariate statistics (5th edition). Pearson Education: Boston; 2007

[17] Gifi A: Nonlinear Multivariate Analysis. John Wiley & Sons, Chichester; 1990

[18] Linting M, Meulman JJ, Groenen PJ, et al: Stability of non-linear principal components analysis: an empirical study using the balanced bootstrap. *Psychological Methods* 2007;12(3):359–79.

[19] Efron B, Tibshirani R: Bootstrap methods for standard errors, confidence intervals, and other measures of statistical accuracy. *Stat Science* 1986;1(1):54–75.

[20] Efron B: Correlation and large-scale significance testing. *J Am Stat Assoc* 2007;107:93–103.

[21] Smylie L: The development and validation of sexual health indicators of Canadians aged 16–24 years. *Public Health Reports* 2013;128(Suppl 1):53-61.

[22] Bell N, Schuurman N, Hayes MV: Using GIS-based methods of multicriteria analysis to construct socio-economic deprivation indices. *Int J Health Geogr* 2007;6:17.

[23] Charlton M, Brunsdon C, Demsar U, Harris P, Fotheringham AS: Principal component analysis: from global to local. 13th AGILE International Conference on Geographic Information Science, Guimarães, Portugal: 2010;1-10.

[24] Psaki SR, Seidman JC, Miller M, et al: Measuring socioeconomic status in multi-country studies: results from the eight-country MAL-ED study. *Popul Health Metr* 2014;12(1):1-11.

[25] Krefis AC, Schwarz NG, Nkrumah B, et al: Principal component analysis of socioeconomic factors and their association with malaria in children from the Ashanti Region, Ghana. Malar J 2010;9:201.

[26] Sharker M, Nasser M, Abedin J, et al: The risk of misclassifying subjects within principal component based asset index. *Emerg Themes Epidemiol* 2014;11:6.
